# Cell surface engineering of *Bacillus subtilis* improves production yields of heterologously expressed α-amylases

**DOI:** 10.1186/s12934-017-0674-0

**Published:** 2017-04-04

**Authors:** Haojie Cao, Auke J. van Heel, Hifza Ahmed, Maarten Mols, Oscar P. Kuipers

**Affiliations:** Department of Molecular Genetics, Groningen Biomolecular Sciences and Biotechnology Institute, University of Groningen, Groningen, The Netherlands

**Keywords:** *Bacillus*, Protein secretion, α-Amylases, Electrostatic interaction, PssA, ClsA, Cardiolipin, Phosphatidylglycerol

## Abstract

**Background:**

*Bacillus subtilis* is widely used as a cell factory for numerous heterologous proteins of commercial value and medical interest. To explore the possibility of further enhancing the secretion potential of this model bacterium, a library of engineered strains with modified cell surface components was constructed, and the corresponding influences on protein secretion were investigated by analyzing the secretion of α-amylase variants with either low-, neutral- or high- isoelectric points (pI).

**Results:**

Relative to the wild-type strain, the presence of overall anionic membrane phospholipids (phosphatidylglycerol and cardiolipin) increased dramatically in the PssA-, ClsA- and double KO mutants, which resulted in an up to 47% higher secretion of α-amylase. Additionally, we demonstrated that the appropriate net charge of secreted targets (AmyTS-23, AmyBs and AmyBm) was beneficial for secretion efficiency as well.

**Conclusions:**

In *B. subtilis,* the characteristics of cell membrane phospholipid bilayer and the pIs of heterologous α-amylases appear to be important for their secretion efficiency. These two factors can be engineered to reduce the electrostatic interaction between each other during the secretion process, which finally leads to a better secretion yield of α-amylases.

## Background

The Gram-positive bacterium *Bacillus subtilis* is one of the best-characterized microorganisms to date. This non-pathogenic cell factory is commonly used for the large-scale production of industrial enzymes due to its genetic amenability and superb fermentation characteristics [1, 2]. The molecular mechanisms underlying protein targeting and export have been studied extensively. Various classical genetic approaches have been applied to enhance gene expression and protein secretion, which has resulted in the development of efficient strains for high-level protein production and recovery [3–5]. However, the expression of heterologous proteins can still be challenging and unpredictable with respect to yield. Efforts to improve our understanding of this economically important process are therefore useful to society and industry [6–8].

Previously, numerous studies have been done to improve the protein production and secretion. For instance, Kakeshita et al. [9] deleted the C-terminus of the SecA secretory machinery to improve the secretion of heterologous proteins. An extracellular α-amylase has been shown to have increased expression in *B. subtilis* by overproduction of PrsA lipoprotein and optimization of regulatory components [10]. In addition, Thwaite et al. [11] has found that the modified cell wall microenvironment (the deficiency of d-alanylation) allows 2.5-folds higher production of recombinant *Bacillus anthracis* protective antigen (rPA). Furthermore, Degering et al. [12] managed to raise the yield of extracellular protease significantly both in *B. subtilis* and *B. licheniformis* by a screening of homologous and heterologous signal peptides. Nevertheless, most of these improvement strategies have focused on the modification of the secretion machinery itself. The engineering of the cell envelope, where secretion takes place, is a novel approach. The cell envelope of *B. subtilis*, which is composed of the lipid bilayer and cell wall, should be transversed by a protein that is excreted by the bacterium into the extracellular environment. Some important aspects of the lipid bilayer and cell wall in relation to secretion are discussed below.

*Bacillus subtilis* has a very complex and variable membrane lipid composition; it consists of 20–50% zwitterionic phospholipid phosphatidylethanolamine (PE), 15–45% phosphatidylglycerol (PG), 2–15% lysyl-phosphatidylglycerol (LysPG), 2–25% cardiolipin (CL) and 10–30% mono-, di- and tri-glucosyl diacylglycerol (GL). The lipid composition changes during growth and cross-regulation between lipid synthesis pathways are suggested to occur in order to maintain membrane functionality and integrity, but how this is regulated is currently unknown [13–15]. The presence of PG in the membrane is essential for the survival of *B. subtilis*, and the specific subcellular localization of SecA in spiral-like structures was shown to have a high PG dependence [16]. Additionally, cardiolipin plays an important role in spore formation [15] and the adaptation to high salt concentrations, and the amount of anionic lipids (PG and CL) in the membrane indicated a strong correlation with the osmo-resistance of the cells [13]. Furthermore, Tat-dependent translocation in *E. coli* was shown to depend on negatively charged phospholipids [17]. Interestingly, the Tat proteins in *B. subtilis* are localized at the poles, where the membrane is enriched in CL. Hence, this lipid might be also important for activity and/or localization of the Tat machinery in *B. subtilis*, but this has not been investigated yet.

The cell wall of *B. subtilis* is a multilayered structure formed by a copolymer of peptidoglycan and anionic polymers (teichoic and teichuronic acid) and contains lipoteichoic acid and proteins. There are two aspects of the bacterial cell wall that can determine the efficiency of passage by a secretory protein, i.e. the charge density and the crosslinking index. Generally, proteins that are translocated via the Sec machinery arrive at the trans-side of the membrane in a relatively unfolded state, where they will encounter the cell wall and are efficiently folded into a protease-resistant conformation [2]. In addition, the overall cell wall net charge is modulated by the extent of d-alanylation of teichoic acid by the products of the *dlt* operon [11]. The inactivation of this operon can increase the net negative charge of the cell wall, thus enhancing the folding and stability of a number of secreted proteins [10, 18]. Besides the charge density, the amount of crosslinking of the thick peptidoglycan layer of the cell wall that determines the size of the holes in the peptidoglycan network, may have significant effects on the efficiency of secretion [19, 20].

In this study, we attempted to weaken the secretion barrier from the cell envelope in order to improve the secretion potential of the *B. subtilis*, realizing that the physicochemical properties of the secreted protein are crucial as well, and the enzyme productivity also depends on the nature of the target protein. The α-amylase from *B. licheniformis* can have a secretion advantage when it is optimized to have a lower isoelectric point (pI) [21]. Knowing this, the cell surface components were genetically engineered and their effects on protein secretion were systematically investigated by using a variety of α-amylases with different pIs. We finally observed that reduced electrostatic interactions increased secretion efficiency of amylase proteins.

## Results

### α-Amylase variants

A variety of α-amylases were chosen as secretion targets in this study. They originate from different *Bacillus* species and were genetically codon optimized. They have either low-, neutral- or high- pI, and were designed and synthesized by DSM and Genencor (Table 1).

**Table 1:** Amylases used in this research

Amylase variants	Organism	Molecular weight (kD)	pI
Amy#707	*Bacillus* sp. 707	56.4	6.72
AmyTS-23	*Bacillus* sp. TS-23	67.3	6.41
AmyBS	*Bacillus subtilis* 168	70.2	5.88
AmyBm	*Bacillus megaterium*	56.5	5.70
AmyK38	*Bacillus* sp. KSM-K38	56.3	4.77

The α-amylase variants have different physicochemical properties. Mature α-amylase proteins (signal sequence cleaved) have molecular masses ranging between 55 and 71 kDa, with pIs ranging between 4.77 and 6.72. They were expected to differentially interact with the cell envelope, finally resulting in a difference of amylase accumulation in the supernatants. They are all publically available and have a good possibility to be expressed and active in parental strain *B. subtilis* DB104. Their secretion capacity was evaluated with respect to the cell surface modification.

### α-Amylases are secreted with different efficiencies in various cell envelope backgrounds

As mentioned previously, the secretory proteins have to get across the two structural hurdles (cell membrane and cell wall) to be released into the medium. During this process, the secreted targets will inevitably interact with various components of the cell envelope. Accordingly, the reduction of this kind of interaction for achieving better secretion potential of the cell factory has a high feasibility.

To begin with, we carefully chose six cell surface relevant enzymes as the modulation targets. Among these candidates, the phosphatidylserine synthase (PssA) and cardiolipin synthase (ClsA) are responsible for the synthesis of two major membrane phospholipids phosphatidylethanolamine (PE) and cardiolipin (CL) [22], respectively. The other four of the candidates all play important roles in cell wall composition and functionality. The teichoic acid linkage unit synthase (TagO) catalyzes the synthesis of wall teichoic acids; the lipid carrier sugar transferase (TuaA) is involved in the teichuronic acids formation; DltA, d-alanyl-d-alanine carrier protein ligase, catalyzes the alanylation of lipoteichoic acid; d-alanyl-d-alanine carboxypeptidase (DacA), mediates the crosslinking of peptidoglycan [20, 23, 24]. A null mutant library (PssA-, TagO-, ClsA-, DltA-, TuaA-, DacA-) was successfully constructed, and the effect of these cell surface alterations on the secretion of target proteins was subsequently analyzed and quantified by enzymatic assays.

**Fig. 1:**
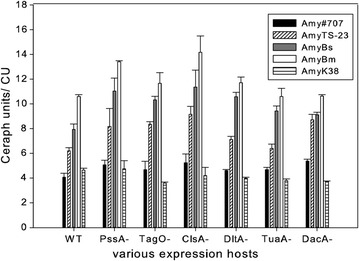
The amylase activity of different variants from various *B. subtilis* deletion mutants and WT. The same OD equivalents of the culture samples were harvested after overnight growing in LB under 37 °C, 220 rpm and the amylase activities were determined by Ceralpha assays. Different patterns of the columns corresponds to various amylases. Each column represents the mean ± SD of three independent experiments, and each assay was performed in duplicate

In comparison with the wild-type strain JMM8901, most of the deletion mutants could significantly produce more α-amylase TS-23, Bs and Bm into the medium, while very low and similar levels of the α-amylases #707 and K38 were secreted from all the hosts, with a yield of around 4 CU/ml (Fig. 1). Among various expression hosts, the ClsA- performed remarkably well on the production of the reporter protein. Compared to WT extracellular concentrations, it generated 47% more of AmyTS-23 and 43% more of AmyBs, while the PssA- strain showed increased production for two of the α-amylase variants (TS-23–32%, BS-39%). In contrast to WT, all of the other four mutants were capable of releasing the elevated amount of AmyBS, and in the TagO- and DacA- background, while AmyTS-23 could also get more than 30% increased extracellular release. To sum up, the enzymatic assay results suggested that inactivation of the aforementioned cell surface components was helpful for excreting the secretory proteins. The PssA- and ClsA- mutants were the two best expression hosts with higher yields of amylases and were prepared for further analysis.

### Specific alteration of cell membrane components and more negative charge of α-amylases could facilitate secretion yields

For further studying the involvement of PssA and ClsA on α-amylase secretion, a double knockout was constructed as previously described. Then, the yield of the above five α-amylases in the three mutant hosts was measured in comparison to WT host JMM8901.

**Fig. 2:**
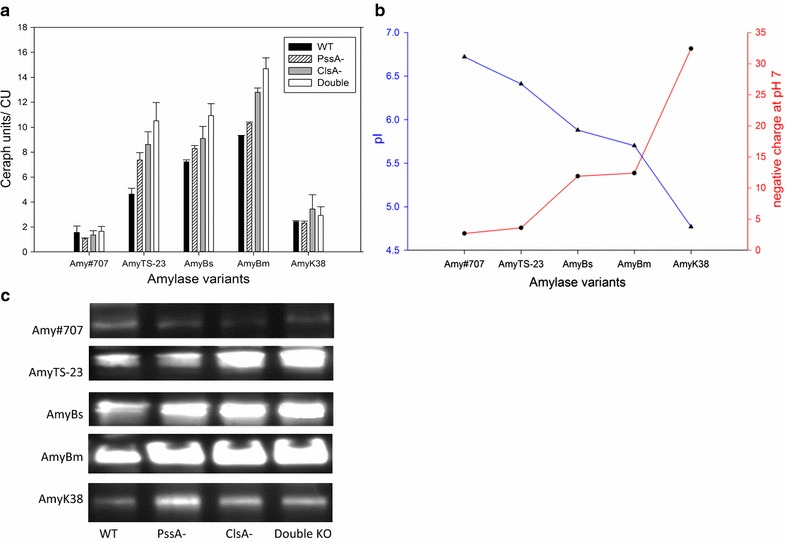
The α-amylase activity in selected expression hosts grown to stationary phase in LB. **a** From *left* to *right*, they are the different α-amylases; for each α-amylase, the *column* pattern represents different hosts. **b** The pI and negative charge of mature α-amylase proteins. The *blue line* represents pIs and the *red line* represents negative charge at pH 7. **c** Western blotting of α-amylase products in the medium using an antibody against *Strep*-tag

The protein samples that were harvested from the medium after induction and expression were subsequently prepared for activity assays and Western blotting. As indicated in Fig. 2a, the accumulation of various α-amylases was quite different in the growth medium, and the α-amylase yields showed a high correlation with the net charge of the target proteins. Generally, the higher negative charge, the more corresponding α-amylases were secreted. This might result from the weaker interaction with the cell surface, as the yields of the α-amylases TS-23, Bs and Bm all went up gradually with the increase of associating negative charge. For the similar reason, the lowest producing α-amylase, Amy#707, had the highest pI and lowest negative charge, and the low production is probably because this α-amylase can strongly interact with cell envelope components, hampering its traverse through. Surprisingly, the secretion of AmyK38, which should be even higher than AmyBm based on pI and charge, turned out to be only a little better than that of Amy#707. This might be owing to the strong binding to some positively charged components or particles causing a severe retardation of AmyK38 in the cell.

Expectedly, when compared with JMM8901, the PssA- and ClsA- strains could significantly enhance the secretion of α-amylases, while the double KO even further improved the increase of protein yields for α-amylase Ts-23, Bs and Bm. In other words, combining these two beneficial modifications would have an additive effect on the α-amylase production. Furthermore, these improvements can also be visualized by protein immunoblotting. As clearly shown in Fig. 2c, from left to right, they are WT, PssA-, ClsA- and double KO, respectively. The α-amylase TS-23, Bs, Bm, were consistently accumulated in the supernatants at an elevated level, while the production of α-amylase K38 and #707 had little difference among different secretion hosts, which suggested a high consistency with preceding enzymatic activity assays. To conclude, the target proteins α-amylase Ts-23, Bs and Bm showed a clear trend for a better secretion in correlation to a higher negative charge, and the double deletion host was the best producer of these α-amylases.

### Anionic membrane lipids go up greatly in the absence of PssA and ClsA

Considering the direct responsibility of ClsA and PssA for the phospholipid synthesis of CL and PE, the single or double deficiency of them can probably alter the lipids composition and cell envelope net charge greatly, which finally resulted in a better performance of the secreted targets. To reveal how the absence of PssA and ClsA influences the phospholipid composition, lipid analysis was performed on four strains (JMM8901, PssA-, ClsA- and the double KO) in stationary phase. The extracted lipid samples from the same amount of various cell pellets were loaded in parallel on the TLC plate. Subsequently, the quantification was done by analysis of the TLC plate with software ImageJ [25].

**Fig. 3:**
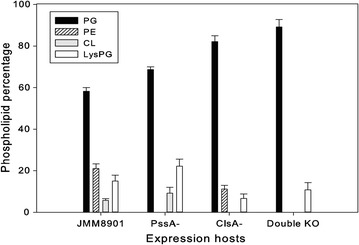
Phospholipids composition of *B. subtilis* wild-type JMM8901, and PssA-, ClsA-, double KO mutant strains in stationary phase. For each strain, the various *column color* represents the different single lipid class, phosphatidylglycerol (PG), phosphatidylethanolamine (PE), cardiolipin (CL) and lysyl-phosphatidylglycerol (LysPG). The values represent the mean ± SD of three independent experiments

The phospholipids analysis demonstrated that all the mutants showed an obvious increase of PG content during the stationary growth phase when compared to its wild-type control JMM8901. The negatively charged phospholipids PG + CL account for 63.9% of total phospholipids in WT. However, the deletion of *pssA* and *clsA* separately raise this percentage to 77.85 and 82.14% respectively. More interestingly, the lack of these two genes in one strain caused an extreme proportion of PG, i.e. almost 90% of the total lipids (Fig. 3). Moreover, the PssA- and ClsA- mutants completely abolished the synthesis of PE and CL respectively, while the double KO resulted in a deficiency of both phospholipids. Taken together, in the null mutants, not only the specific corresponding phospholipid but also others were significantly altered, and especially the increase of the anionic phospholipid PG was observed. This suggests that these modifications could improve the availability of these anionic phospholipids, and thereby affect the electrostatic interactions between protein and cell membrane, and hence influence the rate of protein secretion.

## Discussion

Over the last few decades, numerous attempts have been made to overproduce heterologous recombinant proteins in *B. subtilis*. The conventional approaches have already more or less reached their limits, but there is still a great need for improved heterologous protein secretion systems. *B. subtilis* DB104, a derivative of strain *B. subtilis* 168, which only expresses 4% of the extracellular protease activity in comparison to its parental strain, shows an excellent expression and secretion advantage when being used as a cell factory of industrially important extracellular enzymes [26, 27]. Cell envelope engineering has recently been shown to be beneficial to increase yields in industrial processes, such as the production of enzymes, biofuels and chemicals, and this powerful approach is perfectly suitable for novel protein engineering and directed evolution strategies combined high-throughput screening [28, 29]. Inspired by this, cell envelope engineering is employed in this research to alter the properties of cell surface components with the goal of enhancing heterologous protein production in *B. subtilis* DB104. Moreover, our study is also providing new insights into the critical factors of the secretion process, the pIs of the secreted proteins and characteristics of the cell surface that are crucial for protein secretion efficiency.

The correlation between the cell surface and the secretion ability of the bacterium is rarely known and will be explored by modulating the cell surface composition and functionality. Firstly, we genetically modified the cell surface by the deletion of six selected genes, and the functional consequences on the α-amylase yields had been assessed by enzymatic assays. The enzymatic assays suggest that the absence of ClsA/PssA enhances the α-amylase production, and these beneficial effects can be additive in the double KO. This secretion advantage was strongly suspected as the proper response to the phospholipids composition altering, because previous phospholipid analysis of various isogenic hosts suggested a correlation between anionic lipids and secreted proteins in the supernatants. Consistently, it has been reported that the ClsA- mutant has a higher negative charge density on the cell membrane because of the significant rise of PG [13].

Additionally, the importance of target proteins with specific physicochemical properties in the secretion process was also investigated. The secretion efficiency of α-amylases with different pIs was tested in the various mutant strains and the enzymatic activity assay in combination with Western blotting indicated that the net charge plays an important role in the protein secretion. This led us to the speculation that in general, a higher negative membrane charge, the more proteins get released into the medium. Among the five α-amylase proteins studied, the amyK38 is an exception, with the production level being much lower than expected. That is possibly owing to its net charge and pI being too far away from others, which might either lead to a tight binding to some unknown positively charged intracellular- or cell surface components or to local repulsion by the strong negative charges in the membrane. Surprisingly, despite the relatively small difference between Amy#707 and AmyTS-23 in pIs (6.72 and 6.41) and negative charges at pH 7.0 (2.7 and 3.6), the amylase TS-23 can still be accumulated in the supernatant much more than amylase #707. This might be because of other physiochemical properties of these secreted proteins, or other, yet unidentified factors, influence the overall production yield. In fact, more α-amylases than the above five were investigated in this study. We also engineered different α-amylases with increased positive charge, but unfortunately they showed very low expression and secretion levels, and were very difficult to be detected by our enzymatic assay technique, so we could draw no sound conclusions from this part of the study.

In conclusion, we aimed to improve the use of *B. subtilis* as a cell factory by manipulating and characterizing several factors that influence its protein secretion efficiency. Particularly, we explored the role of electrostatic interactions between the membrane phospholipids and the secreted protein. We managed to design tailor-made *B. subtilis* production strains with enhanced potential for protein secretion, exemplified by various α-amylases, and identifying their pI as an important determinant.

## Conclusions

In *B. subtilis,* during the protein secretion process, the characteristics of membrane phospholipid bilayer and the pIs of heterologous α-amylases determine the electrostatic interaction between the cell surface and secreted proteins and hence influence the secretion efficiency of the α-amylases variants. Consequently, the secretion barrier could be lowered effectively by engineering the cell membrane components and the secreted targets, which finally enhance the secretion yield of α-amylases significantly. In other words, the modification of these two factors provides a large advantage for further improving the protein secretion of *B. subtilis* as a cell factory.

## Methods

### Bacterial strains, plasmids, and medium

All the *B. subtilis* and *E. coli* were grown at 37 °C with shaking (220 rpm) in liquid lysogeny broth (LB). The antibiotics were added when necessary as follows: 100 mg/ml ampicillin for *E. coli,* 5 mg/ml kanamycin, erythromycin, and chloramphenicol, 100 mg/ml spectinomycin, 6 mg/ml tetracycline for *B. subtilis*. For solid medium 1.5% (wt/vol) agar was added to the LB. *B. subtilis* was naturally transformed using Spizizen minimal medium (SMM) supplemented with 0.5% glucose and 50 μg/ml tryptophan [30]. For induction, 0.1% subtilin-containing supernatant of *B. subtilis* ATCC6633 was added for activation of the subtilin-regulated gene expression (SURE) system [31]. The main strains and plasmids used in this study are listed in Table 2.

**Table 2:** The main strains and plasmids used in this study

Strain or plasmid	Genotype or properties	Reference or source
*B. subtilis*		
DB104	*his nprE aprE*	[26]
JMM8900	DB104 *thrC*::*spaRK*, Em^r^	This study
JMM8901	DB104 *thrC*::*spaRK*, Em^r^, *amyE*::Spc^r^	This study
JMM8901-*dacA*	DB104 *thrC*::*spaRK*, Em^r^, *amyE*::Spc^r^, *dacA*::Km^r^	This study
JMM8901-*dltA*	DB104 *thrC*::*spaRK*, Em^r^, *amyE*::Spc^r^, *dltA*::Km^r^	This study
JMM8901-*tagO*	DB104 *thrC*::*spaRK*, Em^r^, *amyE*::Spc^r^, *tagO*::Km^r^	This study
JMM8901-*tuaA*	DB104 *thrC*::*spaRK*, Em^r^, *amyE*::Spc^r^, *tuaA*::Km^r^	This study
JMM8901-*pssA*	DB104 *thrC*::*spaRK*, Em^r^, *amyE*::Spc^r^, *pssA*::Km^r^	This study
JMM8901-*clsA*	DB104 *thrC*::*spaRK*, Em^r^, *amyE*::Spc^r^, *clsA*::Km^r^	This study
JMM8901-*pssA* +*clsA*	DB104 *thrC*::*spaRK*, Em^r^, *amyE*::Spc^r^, *pssA*::Tet^r^, clsA::Km^r^	This study
*E.coli*		
MC1061	F-, *araD*139, Δ(*ara*-*leu*)7696, Δ(*lac*)X74, *galU*, *galK*, *hsdR2*, *mcrA*, *mcrB1*, *rspL*	[36]
Plasmids		
pNZ8901	*PspaSpn repC repA*, Cm^r^	[31]
pNZ8901-Amy#707	pNZ8901 carrying *amy#707*	This study
pNZ8901-AmyTS-23	pNZ8901 carrying *amyTS*-*23*	This study
pNZ8901-AmyBs	pNZ8901 carrying *amyBs*	This study
pNZ8901-AmyBm	pNZ8901 carrying *amyBm*	This study
pNZ8901-AmyK38	pNZ8901 carrying *amyK38*	This study
pUC21	Amp^r^ *lacZ*	[37]
pUC21_DacAKO	pUC21*_dacAUp_*Km^r^ *_dacADown*	This study
pUC21_DltAKO	pUC21*_dltAUp_*Km^r^ *_dltADown*	This study
pUC21_TagOKO	pUC21*_tagOUp_*Km^r^ *_tagODown*	This study
pUC21_TuaAKO	pUC21*_tuaAUp_*Km^r^ *_tuaADown*	This study
pUC21_PssAKO	pUC21*_pssAUp_*Km^r^ *_pssADown*	This study
pUC21_ClsAKO	pUC21*_clsAUp_*Km^r^ *_clsADown*	This study
pUC21_PssAKO(Tet^r^)	pUC21*_pssAUp_*Tet^r^ *_pssADown*	This study

### DNA manipulations

DNA manipulations (purification, digestion, and ligation) were carried out as previously described [32]. T4 DNA ligase, FastDigest restriction enzymes and DNA polymerases (Phusion and DreamTaq) were purchased from Thermo Fisher Scientific (Bremen, Germany). Chromosomal DNA of the *B. subtilis* strain DB104 or plasmids constructed in this research were used as templates for PCR. Oligonucleotides were synthesized from Biolegio (Nijmegen, the Netherlands). Plasmid isolation was performed with the Roche high pure plasmid isolation kit, and all the constructs were sequence-verified by Macrogen Europe (Amsterdam, the Netherlands).

### Construction of the null mutant library

To construct the mutant library, a component of the SURE system, *spaRK* was firstly introduced into the *thrC* locus of parental strain *B. subtilis* DB104 by double recombination, and the obtained strain was named JMM8900. In a similar manner, we replaced the original amylase gene (*amyE*) with Spec^r^ to get the strain JMM8901. Subsequently, a variety of KO plasmids was made as follows: ~1 kb of both upstream and downstream flanking regions were amplified by PCR with Pfux7 DNA polymerase (a kind gift from Bert Poolman, University of Groningen), and the background of plasmid pUC21 and antibiotic resistance genes were also amplified. After that, they were ligated together using the uracil-excision DNA engineering method [33]; the ligation mixture was directly transformed into competent *E.coli* MC1061. Afterward, the positive ones were selected out for deleting corresponding genes in JMM8901, and the target genes (*pssA, tagO, clsA, dltA, tuaA, dacA*) were replaced by an antibiotic resistance gene (Km^r^) separately, and thus a library of single null mutants was successfully obtained. In this way, we made the ClsA + PssA double KO strain as well. All the constructs and strains were verified by PCR and DNA sequencing.

### Construction of the expression vector

**Fig. 4:**
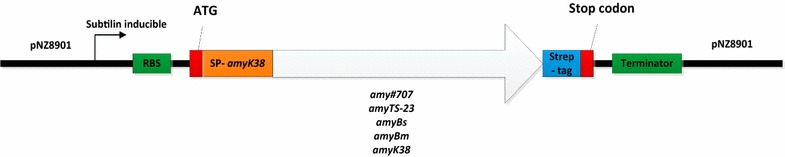
The α-amylase expression vector. The construct contains a subtilin inducible promoter, RBS sequence, signal sequence (SP-*amyk38*), various α-amylase sequences, a *Strep*-tag before the stop codon, terminator, and this whole combination was ligated to the background plasmid pNZ8901

The vector that we used for expression includes a variety of functional components: a subtilin-inducible promoter, a common RBS sequence and terminator, an identical signal peptide sequence of *amyk38*, various amylase-coding regions without the signal peptide, and a *Strep*-tag at the C-terminus, which can be used for further analysis, like protein purification and Western blotting. All these fragments were combined together step by step via overlap PCR and ligation. In this vector, all the factors mentioned above are identical, except the mature α-amylase coding sequences (Fig. 4).

### α-Amylase activity assays

Strains were grown in LB overnight, after which the cells were collected by centrifugation (10,000 rpm, 1 min) and the pellets were suspended and diluted in 200 µl LB (1:50) to start the growth in 96 well plates, the inducer subtilin was added after 3 h’ preculture (1:1000). The optical density was read automatically every 15 min by Infinite 200 plate reader (Tecan, Switzerland) during the whole incubation process. Subsequently, the supernatants were harvested by high-speed centrifugation (14,000 rpm, 1 min) and immediately frozen at –20 °C. The next day, the α-amylase activity was assessed based on the Ceralpha HR Kit manual (Megazyme, Wicklow, Ireland) as follows: 110 µl substrate BPNPG7 solution buffer (54.5 mg BPNPG7 substrate was dissolved in dilution buffer to get 40 ml working substrate) was mixed to 10 µl aliquots of collected supernatant, 140 µl stop buffer (200 mM boric acid/NaOH buffer, pH 10) was added to terminate the reaction after incubating 4 min at room temperature. The tube contents were stirred vigorously before the 405 nm absorbance was read, and the final amylase activity was calculated by the Ceralpha Unit.

### SDS-PAGE and immunoblotting

The same OD equivalents of previously harvested samples were prepared for sodium dodecyl sulfate-polyacrylamide gel electrophoresis (SDS-PAGE) and Western blotting. Firstly, the proteins were separated by SDS-PAGE and transferred onto the polyvinyl difluoro (PVDF) membranes (Millipore, Bedford, MA, USA) using a Mini Trans-Blot system (Bio-Rad) [10], which was then followed by 4 °C overnight incubation in 5% BSA. The next day, the membranes were washed three times 10 min with PBST buffer before incubated with the *Strep*-tag antibody (1:5000; IBA) at room temperature for 90 min, and then the membranes were washed twice with PBST. Finally, the signal density was visualized using the freshly mixed Western blotting detection reagents (GE Healthcare Life Sciences) and detected by the Molecular Imager ChemiDoc XRS+ (Bio-Rad).

### Total lipid extraction and quantification

The extraction of total lipids was performed as reported previously [13, 34]. The same OD equivalents of overnight cultures were harvested by high-speed centrifugation. Then the cells were subjected to sonication for 30 min after washing twice with MilliQ water. Next, the obtained extracts were incubated for 15 min at 54 °C with the same volume of methanol (v/v), followed by the addition of two volumes chloroform (plus 1 ml 6 M HCl to improve the lysPG extraction) [35]. After 16 h’ incubation at room temperature, the mixture was centrifuged for 10 min at full speed. The lower phase containing the lipids was then taken and transferred to a new tube, which was left open and evaporated until dry in the fume hood. The thin-layer chromatography (TLC) with Plate Silica Gel 60 F_254_ (Merck) was carried out for the lipid quantification. The previously dried samples were suspended in 500 μl of chloroform (plus 50 µl 6 M HCl) and then developed with the solvent system acetone/hexane (1:6, v/v) to separate the lipid classes. The purified standards CL, PG, PE, and lysPG, which have been reported as the most abundant lipids in *B. subtilis* membranes were also loaded on the plates. Afterward, the phospholipids were visualized with iodine vapor and analyzed by ImageJ (NIH) software.

## Abbreviations

PssA: phosphatidylserine synthase
ClsA: cardiolipin synthase
DacA: d-alanyl-d-alanine carboxypeptidase
TuaA: lipid carrier sugar transferase
DltA: d-alanyl-d-alanine carrier protein ligase
TagO: teichoic acid linkage unit synthase
SMM: Spizizen minimal medium
SURE: subtilin-regulated gene expression
LB: lysogeny broth
Amp: ampicillin
Spc: spectinomycin
Em: erythromycin
Km: kanamycin
Cm: chloramphenicol
Tet: tetracycline
SDS-PAGE: sodium dodecyl sulfate polyacrylamide gel electrophoresis
PVDF: polyvinyl difluoro
TLC: thin layer chromatography
CL: cardiolipin
PE: phosphatidylethanolamine
PG: phosphatidylglycerol
LysPG: lysyl-phosphatidylglycerol
pI: isoelectric point
